# Maximal Neighbor Similarity Reveals Real Communities in Networks

**DOI:** 10.1038/srep18374

**Published:** 2015-12-18

**Authors:** Krista Rizman Žalik

**Affiliations:** 1 University of Maribor, Faculty of Electrical Engineering and Computer Science, Slovenia

## Abstract

An important problem in the analysis of network data is the detection of groups of densely interconnected nodes also called modules or communities. Community structure reveals functions and organizations of networks. Currently used algorithms for community detection in large-scale real-world networks are computationally expensive or require a priori information such as the number or sizes of communities or are not able to give the same resulting partition in multiple runs. In this paper we investigate a simple and fast algorithm that uses the network structure alone and requires neither optimization of pre-defined objective function nor information about number of communities. We propose a bottom up community detection algorithm in which starting from communities consisting of adjacent pairs of nodes and their maximal similar neighbors we find real communities. We show that the overall advantage of the proposed algorithm compared to the other community detection algorithms is its simple nature, low computational cost and its very high accuracy in detection communities of different sizes also in networks with blurred modularity structure consisting of poorly separated communities. All communities identified by the proposed method for facebook network and *E-Coli* transcriptional regulatory network have strong structural and functional coherence.

Many complex systems in different areas such as sociology^1^, biology^2^, medicine^3^, web^4^ and computer science^5^ can be represented as networks. For example, social networks are represented by people as nodes and their relationships by edges and protein-protein interaction networks and metabolic pathways model biological processes. In most of these networks the nodes are arranged in dense groups that are called communities. Nodes in communities are more densely interconnected to each other than with other communities and generally share common attributes or properties^6^. The identification of community structure helps when analyzing the functionalities and organizations of networks^7^.

Different community detection algorithms have been proposed for identifying natural groups of related nodes within networks^8^. One very popular approach of community detection is the optimization of a modularity function^9^. All algorithms using modularity require knowledge of the whole network and are global algorithms^10^. The use of global algorithms is very difficult for many real-world networks that are huge and with fast changing global structures.

Recently, researchers have proposed several algorithms for detecting communities that optimize some local functions such as local modularity which require the knowledge of local network structure^11^,^12^,^13^. Some fast label propagation algorithms (LP) have been also proposed, where labels propagate at every step when each node adopts the label that most of its neighbors currently have^14^. The label propagation process stops when there are no changes of labels. The main disadvantage of label propagation algorithms is that they produce no unique solutions. They identify different partitions for the same network in multiple runs, while they use some dynamic information: the maximal number of neighbor labels that depends on the processing order of nodes and on some random chosen neighbor labels when there are more equal maximal neighbor labels. In addition the stop criterion of label propagation is only a condition and not a measure that should be optimized. To date, most community detection algorithms have limitations and there is still room for improvement.

In this paper, we investigate a fast and simple algorithm that uses the local network structure and requires neither optimization of pre-defined objective function nor information about number of communities and provides a unique community partition during multiple runs. For finding meaningful communities, the way they are identified is expected to satisfy several basic requirements: it should be based on the density of links, it is required to be local, it cannot be too restrictive, should not yield any cut-node or cut-link and is based on the meaningful definition of communities.

There is no commonly agreed definition of communities. In sociology a community can be a group or network of persons who are connected to each other by social relations to form a tight and cohesive social entity, due to the sharing common values and presence of unity^15^. Many definitions have been proposed during social networks studies^16^, but they are too restrictive or cannot be computed efficiently. Of critical importance in social network analysis is that unit of analysis is not the individual, but at least dyads (two individuals and their ties), triads (three individuals and their ties) or larger subgroups.

Motivated by such perception, the question arose whether the community structure can be uncovered by merging adjacent dyads consisting of a node and its most similar neighbor into communities and using only the maximal cohesiveness of each node with its surrounding. In this paper, instead optimizing some user-defined criteria (e.g. modularity), we consider a community detection from a new point of view: a community consists of pairs of two directly connected nodes- a node and its most similar neighbor. While each node interacts with its local neighbors rather than all other nodes in the network, the local structure of network should be investigated. Observing connectivity pattern of each two nodes of real-world networks gives a natural way to uncover communities. Connectivity pattern influence the closeness and similarity between two nodes.

We propose a community detection algorithm named *kSIM* that starts forming communities with assigning each node to the same community as its most similar neighbor node. The basic observation on which our community definition relies is that a community consists of node pairs of node and its maximal similar neighbor node. Thus, we define a community as a union of tightly connected groups of all adjacent pairs of nodes and its maximal similar neighbors that can be reached from each other through a series of adjacent similar node pairs. Pairs of nodes are adjacent if they share one node, i.e., if they have one common node.

We extended community definition and each node is initially considered to be within the same community as one of its *k* most similar direct neighbors with the smallest degree (number of neighbors). *k* is the parameter that can have values from 1 to the maximal number of neighbors. However, the value *k* = 1 enables to uncover the true real communities for all considered real world networks as also recognized by the other community detection algorithms. *k* greater than 1 enables to detect also small and often overlapped communities.

The proposed *kSIM* method is fast, easy to understand and simple community detection method. It provides the unique solution in multiple runs. The order of processing of nodes do not influence the results. Each node decides to which community it is assigned and the decision requires only local information of the network. The proposed method can also uncover sparse and small communities as well as large and cohesive communities. Community detection algorithms have different approaches offering different balances between speed and accuracy. The advantage of the kSIM approach compared to the other community detection algorithms is its simple nature, low computational cost and its high accuracy in detection communities of different sizes also in networks with blurred modularity structure consisting of poorly separated communities. We analyzed artificial and well-known real-world networks and the resulting partitions are better when compared to those generated by other considered algorithms.

## Results

We tested the performance of our algorithm with artificial networks and the real-world networks with the known community structure. We analyzed 7 different real-world networks shown in Table 1.

### Real-world networks

**Figure 1:**
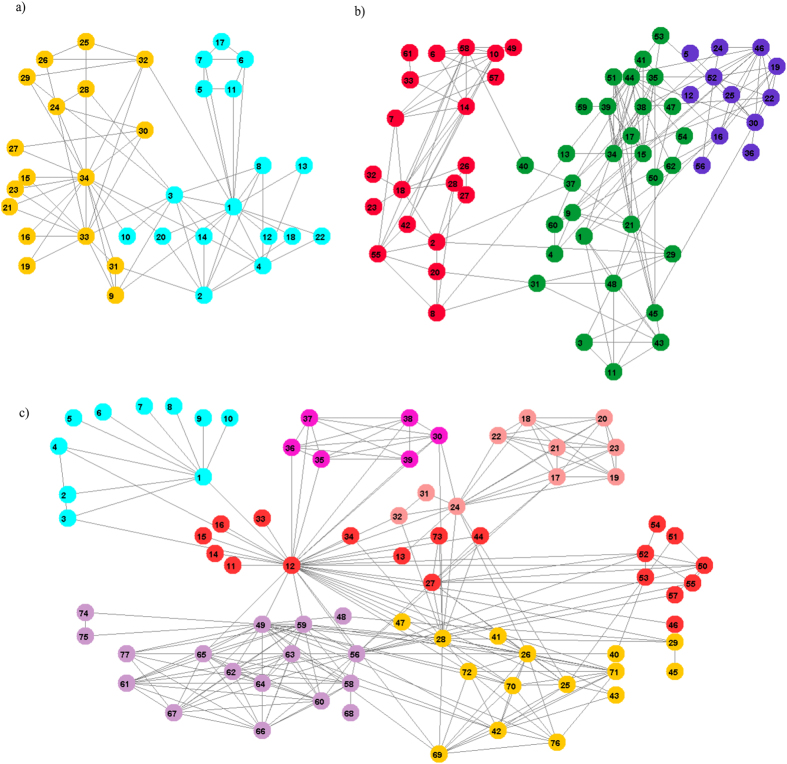
Performance of the proposed kSIM algorithm for parameter *k* = 1 on small real world networks: (**a**) the karate club network and 2 identified communities, (**b**) dolphin network and 3 identified communities (**c**) les Miserables network and 6 identified communities for k = 4. The colors of nodes indicate different detected communities.

The Zachary’s karate club network^17^ consists of 34 nodes, and splits into two smaller clubs after a dispute arose during the course of Zachary’s study. In Fig. 1a two communities obtained by the proposed algorithm kSIM for *k* = 1 are shown.

The Dolphin Social Network describes the associations between 62 dolphins living in Doubtful Sound, New Zealand as reported by Lusseau^18^. In this network the dolphins represented as nodes have links with each other if they are observed together more often than expected by chance during the years from 1994 to 2001. Figure 1b shows the results obtained by the proposed algorithm, where the largest subgroup was spit into two.

The les Miserables data set models the interactions between major characters in the novel Les Miserables, by Victor Hugo, as compiled by Knuth^19^. Each two nodes are connected by an edge only if the corresponding characters simultaneously appear in one or more chapters of the novel. The resulting partition obtained by the proposed algorithm contains 5 communities for *k* = 1, 2, 3 and 6 communities shown in Fig. 1c for *k* > 3. The resulting partition can be described as follows:

- *C*_1_: Community of Bishop Myriel and the people he met; *C*_1_ = {1, 2, 3, 4, 5, 6, 7, 8, 9, 10}.
- *C*_2_: Protagonists of the Champmathieu affair; *C*_2_ = {30, 35, 36, 37, 38, 39}.
- *C*_3_: Community of students and their grisettes (also Fantine-24); *C*_3_ = {17, 18, 19, 20, 21, 22, 23, 24, 31, 32}.
- *C*_4_: The central community with Valjean (12) and the family of Marius; *C*_4_ = {11, 12, 13, 14, 15, 16, 27, 33, 44, 46, 50, 51, 52, 53, 54, 55, 57, 73}.
- *C*_5_: Gavroche, Marius and the revolutionaries and two young children 74, 75; *C*_5_ = {49, 56, 58, 59, 60, 61, 62, 63, 64, 65, 66, 67, 68, 74, 75, 77}.
- *C*_6_: The evil innkeeper Thenardier, his family and accomplices; *C*_6_ = {25, 26, 28, 29, 34, 40, 42, 43, 45, 53, 69, 70, 71, 72, 76}.

**Figure 2:**
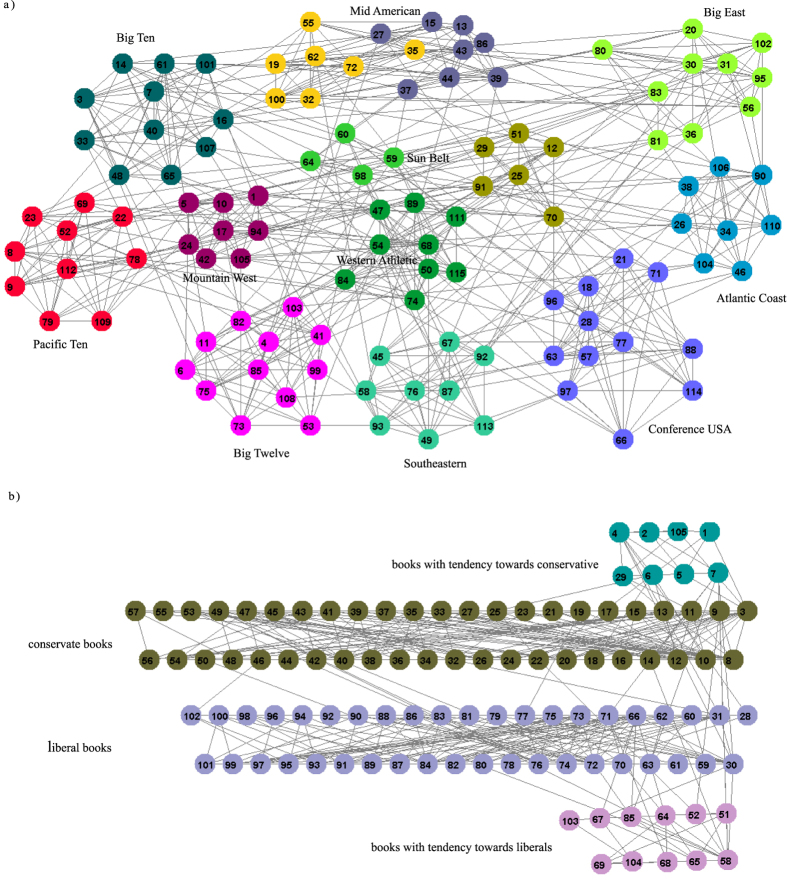
(**a**) US college football network: 9 communities identified by kSIM for k = 1 are equal to 9 real conferences while 2 real conferences are split into two communities; (**b**) the network of books about US politics: four communities identified by kSIM algorithm for k = 1. The colors of nodes indicate different detected communities.

The American college football network^20^ represents the schedule of matches between American college football teams during a single season. The network consisting of 115 teams is divided into 12 groups or conferences, with intra-conference matches being more frequent than inter-conference matches. As Fig. 2a reveals 9 communities are equal to 9 conferences while each of the remaining two conferences are split into two small communities. There are a few independent teams that do not belong to any conference and they are grouped with the conference with which they are more closely associated.

Books about US politics were compiled by Valdis Krebs^21^. The nodes represent 105 books about US politics sold by the on-line bookseller Amazon.com. The edges represent the frequent co-purchasing of books by the same buyers. Books can be divided with respect to the attitude into: liberal, neutral, or conservative. As shown in Fig. 2b the topological structure of neutral is blurred and the algorithm splits it into two small neutral communities with tendencies towards conservative or liberal community. These results discover interesting information, and give us a better understanding of this network.

### Artificial networks

We have evaluated the accuracy of the proposed algorithm kSIM on some artificial networks generated using LFR benchmark^22^ with the following parameters:

- The number of nodes *N* (we set *N* to 5000).
- The average degree *k* with a double bound degrees *kmin* and *kmax* that have been set to 15 and 25.
- The degree of nodes and the size of communities are specified within two power law exponents *β* and *γ* respectively. We have taken *γ* = 2, and 1 < *β* < 2.
- The mixing parameter *μ* indicates the fraction of links connecting each node of a community to nodes in the other communities. The higher value of *μ* means the less modularity structure of a network. We tested with *μ* = 0.1, 0.2, 0.3, 0.4, 0.5, 0.6.

### Result comparisons

**Figure 3:**
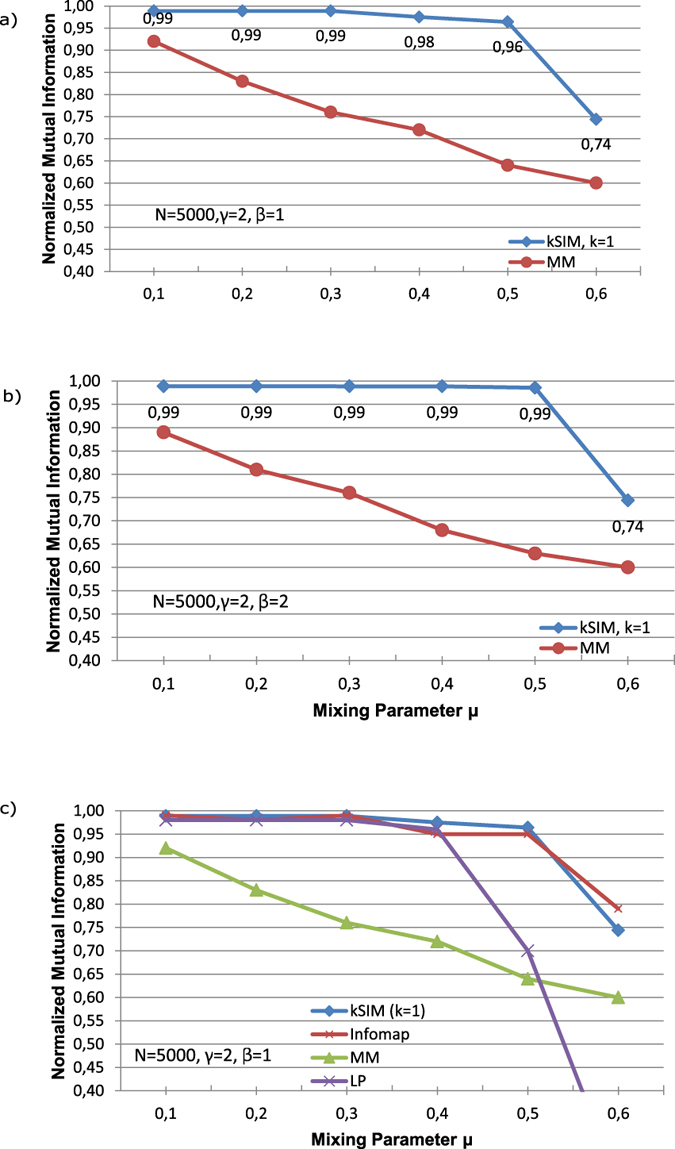
Tests of the proposed kSIM algorithm (k = 1) based on obtained NMI for LFR networks with setting *γ* = 2, N = 5000, *μ* = 0.1, 0.2, 0.3, 0.4, 0.5, 0.6 and (**a**) *β* = 1; (**b**) *β* = 2. Graphs show the values of NMI using kSIM algorithm are greater than using greedy modularity optimization (MM) and so the superiority of kSIM algorithm in finding the higher rate of real communities. (**c**) The values of NMI using kSIM algorithm for LFR network (for setting *γ* = 2, and *β* = 1 and N = 5000) are greater than using greedy modularity optimization (MM) and label propagation (LP) and equal or even better than Infomap for *μ* < 0, 6.

We have tested the efficiency of the proposed kSIM algorithm on various artificial networks LFR and compared them using greedy modularity optimization as shown in Fig. 3. The normalized mutual information (NMI) quality measure was used. It can be seen from Fig. 3a,b, the pure greedy modularity maximization (MM) failed to find a reasonable rate for real communities (*μ* > 0.3, *NMI* < 0.8). Using kSIM method has led to excellent rate in NMI (greater than 0.96 for all *μ* less than 0.6) and consequently has resulted in more accurate community detection for all values of *μ* (see Fig. 3a,b). Mixing parameter *μ* indicates the fraction of links connecting each node of a community to node in the other communities. Thus, the more *μ*, the less modularity structure of a network and the less separated communities. The kSIM method is also efficient in community detection in networks with blurred modularity structure consisting of purely separated communities.

Increasing *β* from 1 to 2 which means having community sizes more similar to each others caused to have 0.03 increase in NMI for some mixing parameter *μ* (0,4 and 0,5) shown in Fig. 3b. For both values *β* = 1 and *β* = 2 the values of NMI are approximately the same and this means that having dissimilarity in community sizes has not had very much effect in accuracy of the algorithm also for networks with blurred modularity structure.

We also compared our method with label propagation (LP) and Infomap method by Rosvall and Bergstrom^23^, while recent comparative analysis of more current community detection methods^24^ concluded that the Infomap is the best performing on the set of benchmarks they have examined in the comparative analysis. Infomap compresses the information of random walk. The optimal compression is achieved by optimizing a quality function, which is the Minimum Description Length of the random walk. Such optimization can be carried out rather quickly with a combination of greedy search and simulated annealing. Infomap is claimed one of the most accurate non-overlapping community detection methods recently. The accurancy of our method is as well or even better (for *μ* = 0.4) as Infomap when *μ* < 0.6, which is the usual case of real-world networks (Fig. 3c).

We tested the proposed algorithm *kSIM* on real-world data sets with known community structures. We compared obtained results by the proposed algorithm *kSIM* to three community detection algorithms: MEP^25^, CNM of Clauset *et al.*^13^ and CFinder^26^. CFinder is based on clique percolation with complexity defined by computational time to identify k-cliques that is an exponentially growing function of the graph size *O*(*n* *exp*(*n*)). Clauset *et al.* (2004) have proposed a fast greedy modularity optimization algorithm (CNM) with algorithm complexity *O*(*n* *log*^2^*n*) on sparse graphs. Starting from a set of isolated nodes, the nodes are iteratively added by CNM such as to produce the maximum possible increase in the modularity. MEP uses some optimization functions and has time complexity of *O*(*n*^2^). We compared the results using three quality measures: modularity Q, MinMaxCut and coverage. The bigger value of modularity and coverage and the smaller value of MinMaxCut denote partition of the higher quality consisting of denser and well-separated communities. Values of all three quality measures for considered real world data sets with known community structure are shown in Table 2. The proposed kSIM algorithm and MEP performed better than the other algorithms. The kSIM algorithm always had stable higher modularity for *k* = 3 and also for *k* = 1. We can see in Table 2 the effectiveness of the proposed algorithm as all quality measurements (also MinMaxCut and coverage) are generally better than MEP, CNM and CFinder. We show that the proposed kSIM algorithm is better or as effective as the other community detection algorithms while it has better time complexity.

### Applications

**Figure 4:**
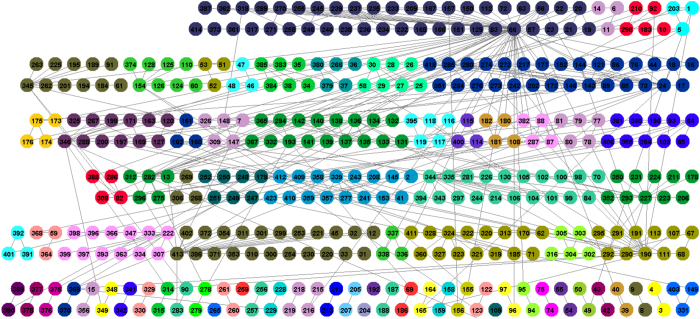
*E. Coli* transcriptional regulatory network: we discovered 55 modules and 5 isolated nodes, 21 communities containing two operons, 9 containing three operons and 25 having more than three operons for k = 1. The colors of nodes indicate different detected communities.

We have applied the proposed method to one bilogical data network forming many small communities and to facebook data set composed of few big communities. We analyzed an undirected version of transcription regulation network *E. Coli* described in database RegulonDB^27^. It is a sparse network with 519 edges and 423 nodes. Nodes are operons consisting of one or more genes transcribed on the same mRNA. Edges go from an operon that encodes a transcription factor to an operon that it directly regulates. Functional modules in the transcriptional regulatory network of *E. Coli* identified by the proposed algorithm are shown in Fig. 4. We identified 5 isolated nodes, 28 communities with two operons, 10 communities with three elements and 25 communities with more than three operons. We analyzed modules with more than 3 elements with the DAVID functional annotation tool^28^ that describes the functional roles of each community’s operons. Therefore, we could measure how well the discovered community structures reflected the real functions. Operons in communities participate in common biological processes. The greater probability that the genes of the operons perform common function is described with smaller p-values. The first cluster with 5 operons (10-ahpCF, 92-dps, 183-gorA, 210-katG, 298-oxyR) participates in anion transport (p-value 5, 10E-44). The third cluster with more than 3 elements (16 − *ansB*, 17 − *appCBA*, 18 − *appY*, 24 − *arcA*, 44 − *aspA*, 73 − *cydAB*, 76 − *cyoABCDE*, 85 − *dctA*, 86 − *dcuB*_*f*_*umB*, 89 − *dmsABC*, 121 − *fdnGHI*, 143 − *fnr*, 144 − *focApflB*. 146 − *frdABCD*, 152 − *fumA*, 177 − *gltA*, 203 − *iclMR*, 217 − *lctPRD*, 242 − *mdh*, 272 − *narGHJI*, 273 − *nark*, 274 − *narL*, 276 − *ndh*, 280 − *nirBDCcysG*, 284 − *nrfABCDEFG*, 285 − *nuoABCEFGHIJKLMN*, 351 − *sdhCDABb0725sucABCD*, 376 − *torR*, 418 − *yjdHG*) performs cellular respiration (p-value 3.1E-82). The fourth cluster with 7 elements (25-argCBH, 26-argD, 27-argE, 28-argF, 29-argI, 30-argR, 58-carAB) performs the biosynthesis process (p-value 8.1E-28). The fifth cluster has 5 elements (36 − *aroH*, 37 − *aroLyaiAarM*, 266 − *mtr*, 379 − *trpLEDCBA*, 380 − *trpR*) and is involved in the aromatic amino acid family bioartificial process RT (p-value2.0E-24).

**Figure 5:**
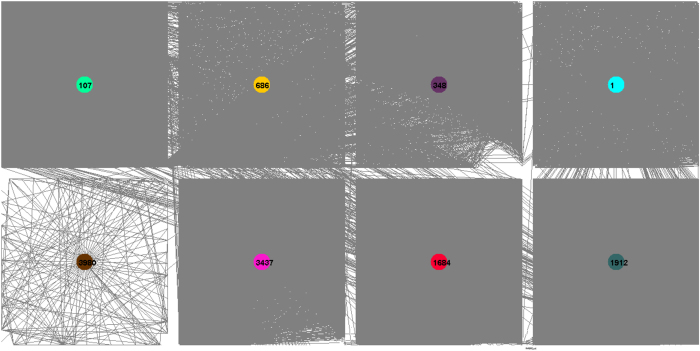
Facebook data set and 8 discovered communities for k = 1. The colors of nodes indicate different detected communities.

The Facebook network consists of circles or friends lists from Facebook^29^. Facebook data was collected from survey participants using Facebook application. The edge indicates that the two users represented by nodes at both ends of the edge are friends. We identified 8 strong sense communities as shown in Fig. 5. Labels of identified communities and numbers of elements in brackets: 1 (666 elements), 107 (1036 elements), 348 (311 elements), 686 (206 elements), 1684 (764 elements), 1912 (751 elements), 3437 (548 elements), and 3980 (56 elements).

## Discussion

In this paper we have proposed a simple and fast community detection algorithm kSIM. It utilizes similarity measure for identifying communities. We have shown the kSIM algorithm identified for *k* = 1 for all considered networks true partitions as recognized also by other community detection algorithms. Using one of the *k*-most similar neighbor nodes for *k* greater than 1 allows to identify smaller communities, thus resulting in higher values of modularity (see Table 2). For the Les Miserables network of novel characters the resulting 7 communities for *k* = 4 are shown in Fig. 1c, while 5 communities are identified for *k* = 1. The parameter *k* can have values from 1 to maximal degree of nodes in networks. The resulting partition depends on *k* but the proposed algorithm provides unique partition in multiple runs of the algorithm for the same value of *k*. Even more, the experiments on the considered networks proved that for more values of *k* the same resulting partition is identified. For example, for the karate club network two communities are uncovered shown in Fig. 1a for *k* = 1 and for *k* > 2 three communities are identified. For the Les Miserables data set 5 communities are identified for *k* = 1, 2, 3 and 7 communities for *k* > 3 and for political books 5 communities are identified for *k* = 3 and 4 communities for all other values of *k*. The parameter *k* is input parameter and it is easy to set not difficult to set as the input parameter number of communities.

The parameter *k* is not difficult to set. The resulting partitions of all considered networks for *k* = 1 are the true partitions as also recognized by other algorithms. For *k* = 3 more communities are identified resulting in higher values of modularity. Quality measurements for *k* = 3 and also for *k* = 1 are generally better than for other compared algorithms.

The forming of communities can be compared to label propagation (LP)^14^. In the kSIM algorithm labels propagate from the more similar neighbors using only static topological information, while in LP the labels propagate based on dynamic information (maximal number of neighbor labels in current iteration) and topological information. This provides unique partition during multiple runs of the kSIM algorithm, while in LP there is more than one distinct partition of a network into groups that satisfies the stop criterion when no label changes. The proposed algorithm has slightly worst time complexity than LP with near linear time complexity. The experiments on various real-world networks demonstrated the efficiency of the proposed algorithm that gives rich information on networks.

Using different *k* in kSIM algorithm usually gives us two or three different partitions. It does not return a set of solution such as some algorithms that formulate community identification as a multi-objective problem and adopt population-based evolutionary algorithms. Multiobjective genetic algorithms for community detection return a set of solution, while each of these solutions corresponds to different values of two objective functions and to diverse partitioning of the network consisting of various numbers of communities^30^. A set of solutions can be also uncovered by the kSIM algorithm with choosing one random neighbor community that give us different resulting partitions over different runs. The experiments proved that multiple community structures can be also uncovered by using the kSIM algorithm and assigning the node within the same community as its *n*–*th* greatest similar neighbor node or the least similar if *n* is greater than the number of neighbor nodes.

**Figure 6:**
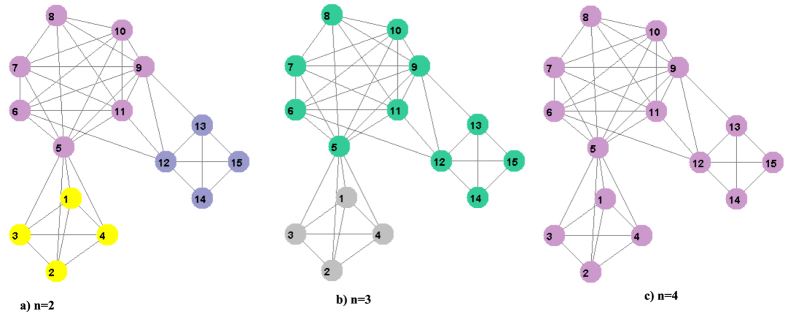
Multiple solutions for artificial network obtained by using different n-th greatest neighbor nodes: (**a**) n = 2, (**b**) n = 3, (**c**) n = 4. The colors of nodes indicate different detected communities.

Using the *n*–*th* greatest similar neighbor gives multiple partitions for the artificial networks shown in Fig. 6a–c. Note that the solutions have the hierarchical structure. For example, the partition in Fig. 6b consists of communities obtained by merging of the two communities of partition in Fig. 6a and in Fig. 6c all communities are merged into one.

The overall advantage of the kSIM algorithm compared to the other community detection algorithms is its simple nature, low computational cost and its high accuracy in detection communities of different sizes also in networks with blurred modularity structure consisting of poorly separated communities. The kSIM algorithm identified communities in the considered artificial networks not only with more value of modularity, but also more accurately in terms of higher NMI values. Its low cost and good accuracy enables the proposed algorithm to be applied on possibly very large networks.

## Methods

We have defined community based on two criteria and then propose a simple framework to detect communities based on network topology using local network information. Nodes of communities have to satisfy membership criteria and community criteria is used to define communities.

### Community criteria

Community structure is the tendency for nodes to divide into groups, with dense connections within groups and only sparse connections between them^31^,^32^. So communities have larger internal connections than external. However, this definition is unable to detect a dense central community with little less dense surrounding. An example of such network is in Fig. 5 in supplementary materials. Communities within social networks are such dense subnetworks surrounded with less dense neighborhoods. People often have many more external relationships, weak or even strong, than they have with the local group to which they belong. Nevertheless including these relationships should not disable any algorithm from identifying natural communities. To identify such dense communities with little less dense surroundings then each community should have more internal connections than external connections to any other community.

To maximally increase the ratio of internal to external connections, each node has to be assigned to the same community as one its most similar neighbor node with maximal common neighbors. The community consists of groups of adjacent node pairs that have more internal connections than external connections to any other community. Node pairs consist of node and one of its *k* most similar direct neighbors. Adjacent pairs of nodes have one common node.

### Membership criteria

Each node as a member of a community has to maximally increase the ratio of internal to external connections. We have defined membership criteria that requires nodes to be within the same community as one neighbor node from the *k* most similar direct neighbors (with higher number of common neighbors). If there are more neighbors with the similar value of similarity the neighbor with the smallest node degree (number of neighbors) is chosen. We used different similarity indexes explained in the supplementary material.

That is to say, each node and one neighbor node from the *k* most similar direct neighbors with the lowest node degree should be in the same community, except for overlapping nodes. Overlapping nodes are on the periphery of each community and have connections to other communities. Each node by definition should have more neighbors within its community than with any other community.

### Algorithm

The proposed algorithm *kSIM* consists of seven steps: calculating similarities between nodes, identifying pairs of nodes with searching the maximal similar neighbor nodes of each node, detecting communities of adjacent pairs, and while there are changes reassigning nodes to satisfy membership criteria and merging communities that do not satisfy community criteria and two output steps: output isolated communities and output all detected communities. The kSIM algorithm requires less then 4 iterations for the considered data sets shown in Table 1. The detail description of algorithm and an example of the application of the algorithm to a network is shown in the supplementary materials.

#### Evaluation measures

We used the following four quality measures for evaluating the effectiveness of the proposed algorithm: modularity, coverage, MinMaxCut and NMI. Modularity is an often used quality measure proposed by Newman and Girvan^20^. In modularity, the number of edges inside communities is compared to the expected number of edges in the randomized network within the same node degree. Modularity *Q* (Eq. 1) is the sum of the differences regarding the fractions of all edges of the *i*th community *e*_*ii*_ and expected fractions of edges within group 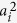. 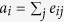 where *e*_*ij*_ is the fraction of edges between groups *i* and *j*. Maximizing modularity *Q* leads to dense and well-separated communities.


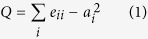


The MinMaxCut of a partition P (Eq. 2) is the fraction of *e*_*ii*_ - the number of edges of community *C*_*i*_ compared to the number of edges between *C*_*i*_ and other communities *e*_*i*′_^33^. Minimizing the MinMaxCut leads to well-separated and dense communities.


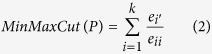


The coverage of a partition P is defined as the fraction of interior edges *e*_*ii*_ in the communities compared to the total number of edges *E*^34^. Maximization of the coverage in Eq. 3 produces dense communities.


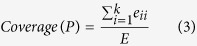


In order to evaluate the performance of our algorithm in detecting artificial networks we used the normalized mutual information (NMI) measure^35^ to evaluate community partitions. NMI is defined as follows:


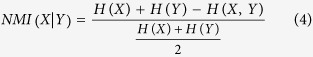


where X corresponds to the real communities, Y corresponds to the predicted communities, H(X) denotes the entropy of community X, and H(X,Y) denotes the joint entropy of X and Y. Normalized mutual information *NMI* is calculated from mutual information *I*(*X*; *Y*)


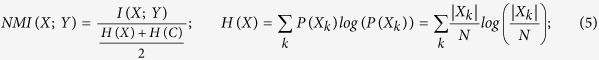



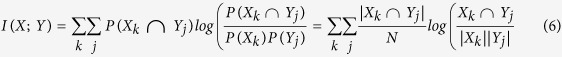


where *P*(*X*_*k*_), *P*(*Y*_*j*_), 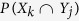 are the probabilities of individuals being in community *X*_*k*_ or *Y*_*j*_ and in the intersection of *X*_*k*_ and *Y*_*j*_ respectively.

### Complexity Analysis

Community detection algorithms have to have low time complexities to be applied to large-scale networks. Our algorithm consists of calculating similarities, forming of communities and adjusting communities and nodes among communities. Calculation of similarities requires *O*(*d*^2^*n*) where *n* is the number of nodes and *d* is the maximal number of node neighbors (node degree). Choosing one similar node of the *k*-most similar neighbors costs *O*(*kn*). Then communities can be formed in *O*(*m*) time complexity, where *m* is the number of edges within the network. Adjusting communities and nodes among communities requires *O*(*vK*^2^ + *sK*^2^ + *Kn*) where *K* is the number of communities, *v* is the maximum number of overlapping nodes between two communities and *s* is the maximal size of the community. The total time complexity of the proposed algorithm is: *O*((*v* + *s*)*K*^2^ + (*K* + *d*^2^)*n* + *m*).

## Additional Information

**How to cite this article**: Žalik, K. R. Maximal Neigbour Similarity Reveals Real Communities in Networks. *Sci. Rep.* **5**, 18374; doi: 10.1038/srep18374 (2015).

## Supplementary Material

Supplementary Information

## Figures and Tables

**Table 1:** Datasets used in experiments and the number of iterations required by the proposed kSIM algorithm.

dataset	nodes	edges	communities	description	iterations k = 3
Karate	34	78	2	Zackary’s karate club	2
Dolphins	62	159	2	Dolphin social network	2
Les Miserables	77	254	6	Coappearance of characters in the novel Les Miserables	1
Football	115	616	11	US college football	3
Polbooks	105	441	3	Books about US politics	2
*E. Coli*	423	519	21	Transcriptional regulation data	3
Facebook	4039	88218	10	Facebook network	3

**Table 2:** The analyzes of considered real world networks using three quality measurements as described in the Section Evaluation measures, where the bigger value of modularity and coverage and the smaller value of MinMaxCut denote partitions of higher quality consisting of denser and well-separated communities.

data set	Quality measure	CNM	Cfinde	MEP	kSIM(*k* = 1)	kSIM(*k* = 3)
Karate	Modularity	0.380	0.183	0.371	0.372	0.402
Karate	MinMaxCut	0.52534	4.983	0.588	0.588	1.544
Karate	Coverage	0.756	0.936	0.872	0.872	0.821
Polbooks	Modularity	0.501	0.491	0.526	0.524	0.526
Polbooks	MinMaxCut	4.618	5.904	2.005	2.118	2.013
Polbooks	Coverage	0.919	0.735	0.911	0.909	0.907
Les Miserables	Modularity	0.500	0.436	0.471	0.501	0.538
Les Miserables	MinMaxCut	4.279	8.050	1.406	4.644	3.978
Les Miserables	Coverage	0.732	0.677	0.862	0.803	0.795
Football	Modularity	0.577	0.596	0.601	0.580	0.602
Football	MinMaxCut	5.551	18.757	14.128	17.092	14.088
Football	Coverage	0.742	0.688	0.692	0.664	0.692
