# Supplementary material for: Maximal Neighbor Similarity Reveals Real Communities in Networks
Source: Sci Rep. 2015 Dec 18;5:18374. doi: 10.1038/srep18374 (PMC4683394; doi:10.1038/srep18374)
Supplement: Supplementary Information [file srep18374-s1.pdf]

Maximal Neighbor Similarity Reveals Real Communities in Networks

Krista Rizman Žalik

In order to explain the proposed community detection algorithm named kSIM, we provide a detailed description of each step using the Karate club network in Section 2. Basic definitions of graphs and used node similarity measures are described in Section 1.

Basic definitions of graphs and node similarity measures

A network can be represented as a graph $G(V,E)$ with a set of nodes $V=(v_1,v_2,..v_n)$ that models objects and a set of edges E that models relationships between each pair of nodes that interact within complex systems or they are just similar enough. An edge describes a certain degree of interaction between two nodes. Communities are defined as natural groups of densely interconnected nodes that are only sparsely connected to the rest of the network. Partition $P=C_1\bigcup C_2\bigcup ...\bigcup C_m$ consist from m communities $C_1,C_2,...C_m$ that can be disjointed or can overlap. Given an undirected graph G, the neighborhood of a node $u\in V$ is the set Γ_u containing adjacent nodes of node u.

$$\Gamma_u=\{v\in V|\{u,v\}\in E\} \tag{1}$$

Common neighbors of two nodes $u,v\in V$ is the set Γ_{uv} containing adjacent nodes of node u that are also adjacent nodes of node v.

$$\Gamma_{uv}=\{c\in V|\{u,c\}\in E \ and\ \{c,v\}\in E\} \tag{2}$$

Nodes are connected with its neighborhood. The connectivity pattern of each two nodes of real-world networks gives a natural way to uncover communities. Indirectly connected nodes with some common neighbors (nodes x and y in Fig. 1a) are less similar than directly linked nodes (nodes x and y in Fig.1b). Like a friendship network, each person affects their known people. More common known people make two acquaintance more close to one another. But on closeness of two strangers (without direct connection in a network) more common known people have no influence. Two nodes x and y are more similar to one another when they have more common neighbors (nodes x and y in Fig 1c). Neighbors that exclusively belong to only one node of two connected nodes do not influence the closeness and similarity of these two nodes (nodes x and y in Fig. 1d). Theoretically, once all neighbor nodes of two nodes are common then such two nodes can be viewed as the same node (nodes x and y in Fig. 1e).

The similarity measure based on common neighbors can model the proposed interaction model. We use the similarity measure for each pair of nodes i and j named $CommonNeigbors_{ij}$ that is the sum of number of links among nodes i and j and the number of common neighbors of nodes i and j:

$$CommonNeigbors_{ij}=A_{ij}*(1+\Gamma_{ij})=A_{ij}*(1+\sum_{k\in V}A_{ik}*A_{jk}) \tag{3}$$

where A is the adjacency matrix. A network $G(V,E)$ can be represented by a $n*n$ adjacency matrix $A=[A_{ij}]$ where $A_{ij}=1$ if there is an edge between node i and node j and $A_{ij}=0$ otherwise.

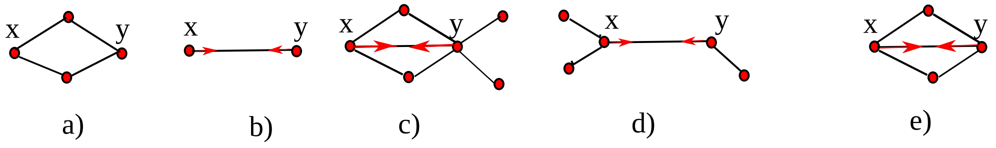

Figure 1. Illustration of closeness (similarity) of nodes influenced by different interaction patterns: a)unlinked nodes x and y; b) direct neighbors x and y; c)directly linked nodes that have some common neighbors; d) directly linked nodes that have exclusive neighbors; e) directly linked nodes x and y without exclusive neighbors that can be viewed as the same node.

Several node similarity metrics on the bases of local information have been proposed. [1] The node similarity measures used in this paper are Salton's Cosine index[2] and the Jaccard index.[3] The Jaccard index is defined as the ratio of common neighbors of nodes i and j, normalized by the sum of the neighbors of both nodes:

$$JaccardIndex = \frac{|\Gamma ij|}{|\Gamma i| + |\Gamma j| - |\Gamma ij|} \tag{4}$$

Salton's Cosine is computed as the ratio of objects contained within communities i and j, normalized by the square root of the product of the objects from communities i and j:

$$CosineIndex = \frac{|\Gamma ij|}{\sqrt{|\Gamma i| * |\Gamma j|}} \tag{5}$$

The proposed method is based on the following:

(i) At the beginning each node is considered as a community. Each node is assigned a unique label and the same label is assigned as the community label denoting the community to which the node belongs.

(ii) The proposed community algorithm is based on the idea that each node belongs to the same community as its most similar direct neighbor. We start by identifying the maximal similar direct neighbors and assigning them in the same communities. The similarity between each node and all its direct neighbors are calculated using one similarity measure (Eq.2-4).

(iii) When a node has two or more direct neighbors belonging to different communities with the same similarity values then we have the following two possibilities of choosing the community of the current node.

1. possibility: We can assume that the node tends to group with its neighbor node that has maximal degree (number of its neighbors) by analogy to the preferential attachment mechanism proposed by Barabasi and Albert in scale-free evolving network.[4] Preferential attachment means that the more connected a node is, the more likely it is to receive new links. Nodes with higher degrees have stronger abilities for grabbing links added to the network.

2. possibility: We can assume that the node tends to group with its neighbor node with minimal degree (number of its neighbors), so that small communities with low densities can also be identified.

We used the second possibility and extended the method to use the neighbor community of one of the k most similar direct neighbors with the lowest degree for assigning a node to the community.

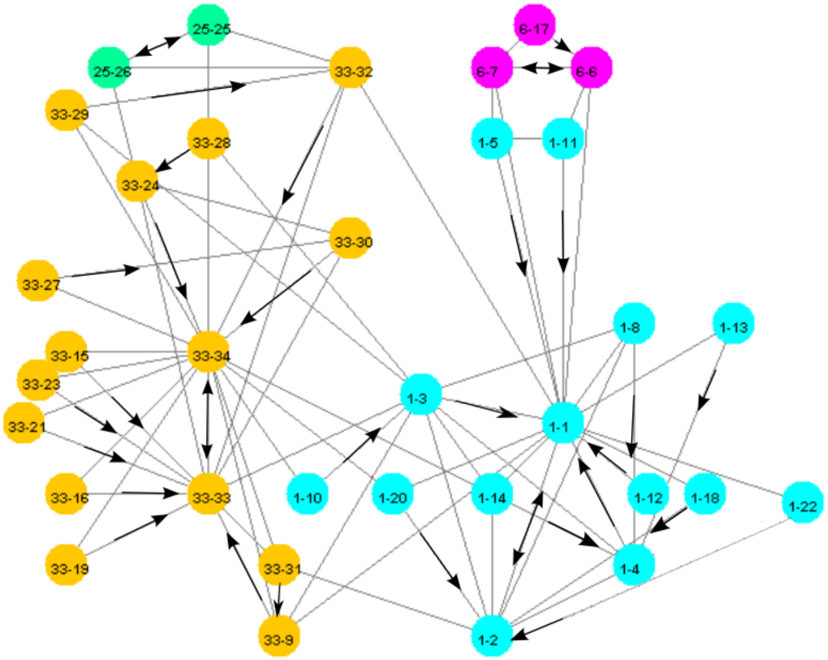

Figure 2. Identified preliminary communities in the karate club network using one most similar direct neighbor after the first phase of the algorithm; from each node one arrow points to the most similar direct neighbor node. The first number in each node is community label and the second number is node label.

Table 1. Nodes and their most similar direct neighbors for the Karate data set. The values of CommonNeigbors index (Eq. 3) are in brackets.

v node	the most similar neigbor *n* ($CommonNeigbors_{vn}$)	2.most similar neigbor *m* ($CommonNeigbors_{vm}$)
1	**2**(8)	3(6) ,4(6)
2	**1**(8)	3(5),4(5)
3	**1**(6)	2(5),4(5)
4	**1**(6)	2(5),3(5)
5	**1**(3)	7(2),11(2)
6	**7**(3)	1(3)
7	**6**(3)	1(3)
8	**4**(4)	2(4), 3(4), 1(4)
9	**33**(4)	31(3), 34(3)
10	**3**(1)	34(1)
11	**1**(3)	5(2),6(2)
12	**1**(1)	=
13	**4**(2)	1(2)
14	**4**(4)	1(4), 3(4), 2(4)
15	**33**(2)	34(2)
16	**33**(2)	34(2)
17	**6**(2)	7(2)
18	**2**(2)	1(2)
19	**33**(2)	34(2)
20	**2**(2)	1(2)
21	**33**(2)	34(2)
22	**2**(2)	1(2)
23	**33**(2)	34(2)
24	**34**(4)	30(3), 33(3)
25	**26**(2)	32(2)
26	**25**(2)	32(2)
27	**30**(2)	34(2)
28	**24**(2)	34(2)
29	**32**(2)	34(2)
30	**34**(4)	33(3), 24(3)
31	**9**(3)	33(3), 34(3)
32	**34**(3)	33(2), 25(2) , 26(2), 29(2)
33	**34**(11)	9(4)
34	**33**(11)	24(4), 30(4)

The proposed method

In order to make our method clearer to readers, we provide a detailed description of the steps of community detection regarding the Zackary karate club,[5] that is a real-world network consisting of 34 nodes. The flowchart of our algorithm is shown in Fig. 4. The community detection process consists of the following steps:

Step 1: Calculate similarities. According to Eq. 3, the similarities between each two connected nodes of the input network are calculated.

Step 2: Find the most similar neighbor among k-maximal similar neighbors for each node. In this example we set parameter k=1 and so we used one maximal similar neighbor. For each node of the karate network, we identified the most similar neighbor among the neighbors presented in Table 1. When there were more maximally similar neighbors we chose the one with the smallest number of its neighbors (node degree). For example node 14 has four neighbors (4,1,3,2) with maximal number of common neighbors (3) and one of them with the lowest node degree (node 4) is chosen as the most similar neighbor. At the end of this step we have one most similar neighbor for each node.

Step 3: Detect preliminary communities. Preliminary communities are all separated parts of the graph with all nodes of input network and edges connecting each node with its most similar neighbor node. If two or more neighbor nodes are equally similar the one with the smallest degree (number of neighbors) is chosen. The identified preliminary communities for the

Zackary karate club are shown in Fig 2. Each arrow point to the chosen (the most similar) direct neighbor with the same community label. Four candidate communities are formed for the karate network, two bigger communities labeled as 1 and 33 and two small labeled as 25 and 6, respectively.

Step 4: Output isolated communities that have no external links and so no connections with other communities. Detected preliminary communities that have no connections with other communities cannot change any more during the following steps of the algorithm. They are parts of the resulting partition. If they contain one or a small number of nodes then they are outliers.

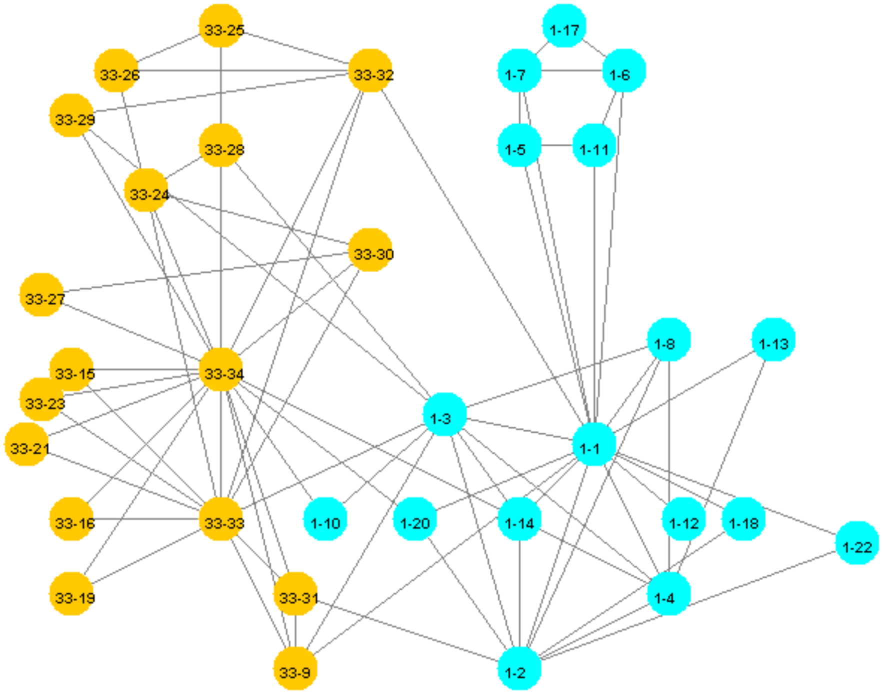

Figure 3. Karate club network divides into two communities. The first number in each node is community label and the second number is node label.

Step 5: Verify the detected communities. Each community that does not satisfy community criteria merges with the community with which it has the most connections. If there are two neighbor communities with the same number of connections, the community with the smaller number of elements is chosen. In the Karate network, the community 6 is merged with 1 and 25 with 33. All identified communities are shown in Fig. 3.

Step 6: Adjust overlapping nodes and assign them to the community with which they have the most neighbors. For each member of the candidate community, compute the connections between it and every candidate community, and assign it to the community which has the most connections with it. First, the nodes are processed from the biggest community and then at last from the smallest. In this example, no node is misallocated and no node is reassigned. Each overlapping node that has equal maximal direct neighbors with two or more communities is assigned to the community of its direct neighbor with the smallest degree. In this case, node 10 is an overlapping node with the same number of direct neighbors in two communities. It is assigned to community 1.

Repeat steps 5 and 6 until there are changes.

Step 7: Output all detected communities.

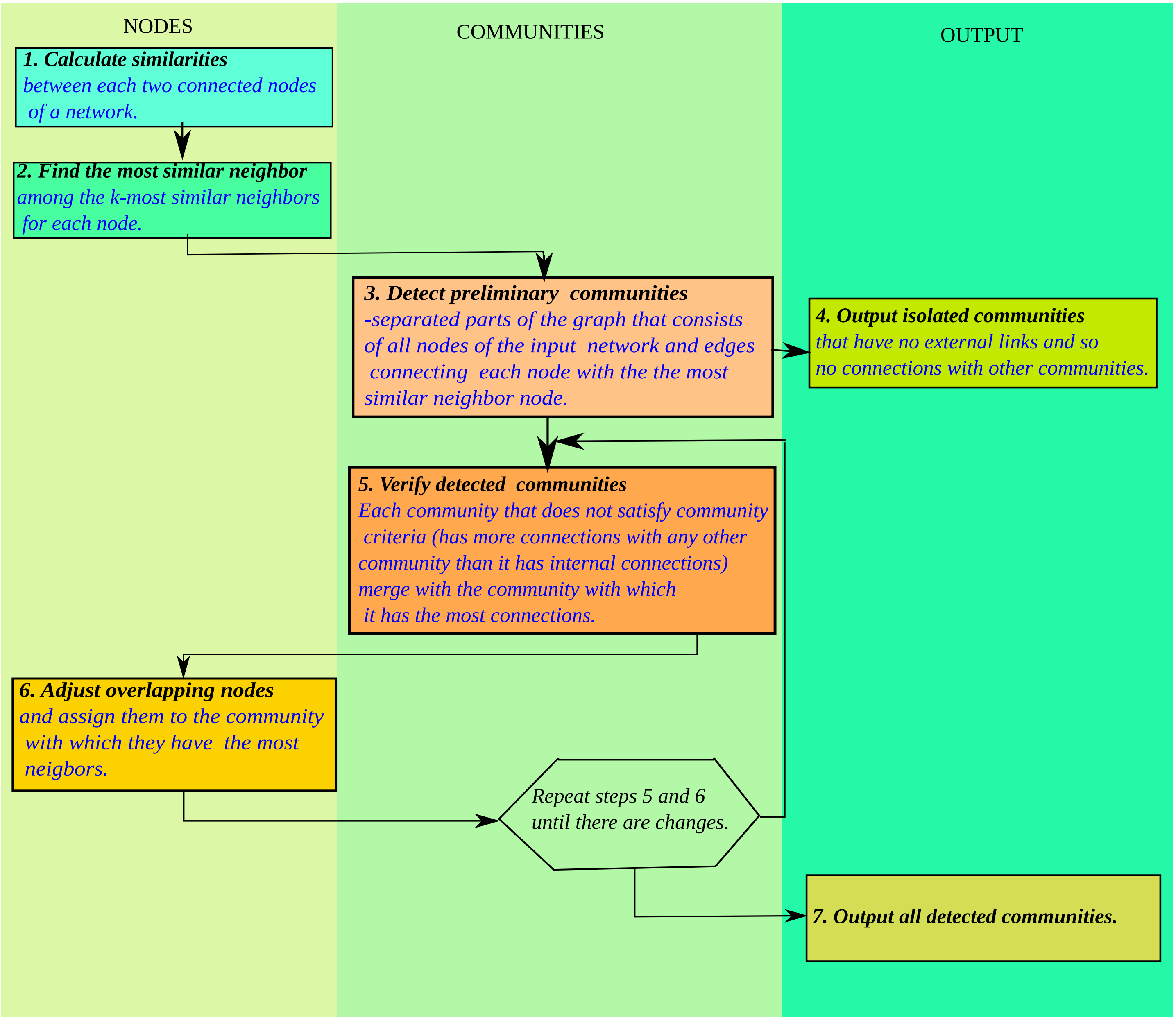

Figure 4. Activity diagram describing the proposed kSIM algorithm with 7 steps.

The algorithm can also detect a dense central community with little less dense surrounding as shown in an example network in Fig. 5.

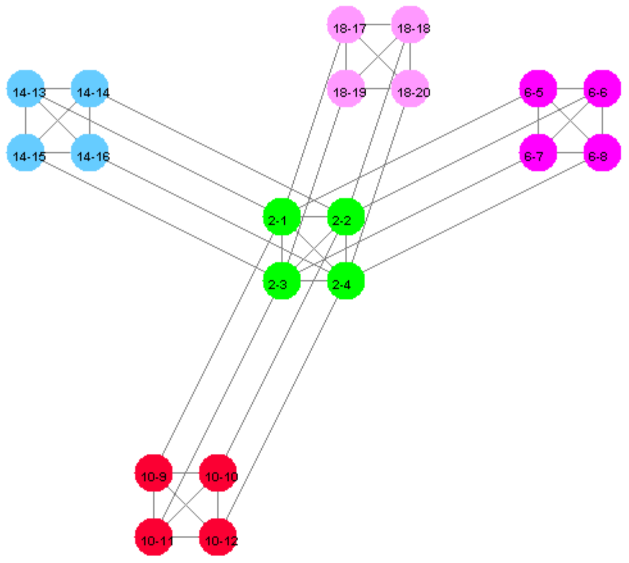

Figure 5. An example network and identified communities for k=1. The first number in each node is community label and the second node label.

Using k most similar direct neighbors

Using k most similar direct neighbors with k greater than 1 allows the uncovering of also small communities with low densities as shown on a small example network in Fig. 6. Using one most similar neighbor (k=1) results in the two identified communities shown in Fig. 6.a while using two or more more similar neighbors ($k >= 2$) identifies the three communities shown in Fig. 6.b. The vertex 12 has the most similar neighbor vertex 9 that causes forming of big community with label for $k = 1$. For $k >= 2$ the community label of the neighbor 13 is chosen while it has the smaller number of neighbors than nodes. The first three most similar nodes of the node 12 and values of *CommonNeigbors* index in brackets are: 9(5), 13(4), 6(4), 15(4),11(4).

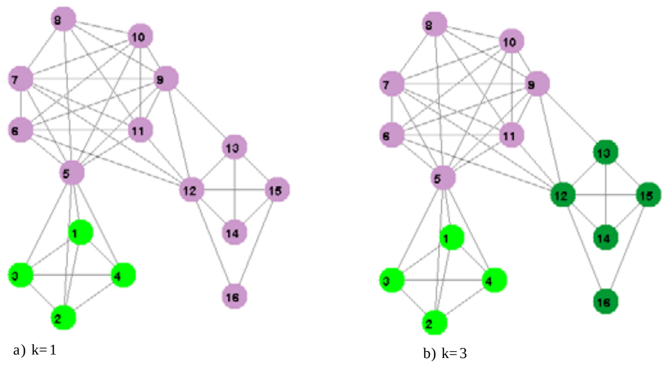

Figure 6. Identified communities of the example network: a) using k=1 - one most similar direct neighbor and b) using k=3. The colors of nodes indicate different detected communities.

Using different similarity measures

We performed experiments with all considered data sets and all three considered indices. The results in Table 2 show that kSIM algorithm using the CommonNeigbors index identified the true real partitions, while Jacard and Cosine index identified also more small communities that were also uncovered using CommonNeigbors index and parameter $k = 3$ for considering three most similar neigbors.

Table 2. The number of discovered communities for the considered datasets discovered by the proposed kSIM algorithm using different similarity indexes and parameters.

dataset	CommonNeigbors k=1	CommonNeigbors k=3	Jacard index	Cosine index
example	2	3	3	2
Karate	2	3	3	2
Dolphins	3	4	4	4
Les Miserables	5	7	6	6
Football	13	12	12	12
Polbooks	4	4	4	4
Facebook	8	22	22	22

References

1. Zhou,T., Lu,L., Zhang, Y.C. Predicting missing links via local information. *Eur. Phys. J. B* **71**, 623-630 (2009).

2. Salton, G., Macgill, M. J. *Introduction to Modern Information Retrieval*. McGraw-Hill. (1983).

3. Jaccard, P. Etude comparative de la distribution florale dans une portion des Alpes et des Jura. *Bulletin del la Societe Vaudoisedes Sciences Naturelles* **37**, 241-272 (1991).

4. Barabasi, A.L., Albert, R. Emergence of scaling in random networks. *Science* **286**, 509- 512 (1999).

5. Zachary, W. W. An information flow model for conflict and fission in small groups. *Journal of Anthropological Research* **33**, 452-473 (1977).
